# A Sacrificial PLA Block Mediated Route to Injectable and Degradable PNIPAAm-Based Hydrogels

**DOI:** 10.3390/polym12040925

**Published:** 2020-04-16

**Authors:** Vernon Tebong Mbah, Vincent Pertici, Céline Lacroix, Bernard Verrier, Pierluigi Stipa, Didier Gigmes, Thomas Trimaille

**Affiliations:** 1 CNRS, Aix-Marseille University, ICR UMR 7273, 13397 Marseille, France; t.b.vernon@pm.univpm.it (V.T.M.); pertici.vincent@hotmail.fr (V.P.); didier.gigmes@univ-amu.fr (D.G.); 2 Dipartimento di Scienze e Ingegneria della Materia, dell’Ambiente ed Urbanistica, Università Politecnica delle Marche, via Brecce Bianche, 60131 Ancona, Italy; p.stipa@staff.univpm.it; 3 CNRS, Université Claude Bernard Lyon 1, UMR 5305, Biologie Tissulaire et Ingénierie Thérapeutique, IBCP, 69367 Lyon, France; celine.fish@free.fr (C.L.); bernard.verrier@ibcp.fr (B.V.)

**Keywords:** block copolymers, poly(N-isopropylacrylamide), polylactide, thermoresponsive, injectable hydrogel

## Abstract

Thermoresponsive poly(N-isopropylacrylamide) (PNIPAAm)-based injectable hydrogels represent highly attractive materials in tissue engineering and drug/vaccine delivery but face the problem of long-term bioaccumulation due to non-degradability. In this context, we developed an amphiphilic poly(D,L-lactide)-b-poly(NIPAAm-co-polyethylene glycol methacrylate) (PLA-b-P(NIPAAm-co-PEGMA)) copolymer architecture, through a combination of ring-opening and nitroxide-mediated polymerizations, undergoing gelation in aqueous solution near 30 °C. Complete hydrogel mass loss was observed under physiological conditions after few days upon PLA hydrolysis. This was due to the inability of the resulting P(NIPAAm-co-PEGMA) segment, that contains sufficiently high PEG content, to gel. The copolymer was shown to be non-toxic on dendritic cells. These results thus provide a new way to engineer safe PNIPAAm-based injectable hydrogels with PNIPAAm-reduced content and a degradable feature.

## 1. Introduction

In the last decade, injectable hydrogels (i.e., liquid at room temperature and in situ gelling at physiological one) have become highly desirable biomaterials in tissue engineering and drug/vaccine delivery due to their minimally invasive character [1,2,3,4,5,6]. These hydrogels can be either chemically cross-linked using multi-functional precursors to be clicked [7,8,9], or physically cross-linked through non-covalent interactions (H-bonding [10], ionic interactions [11,12]), or thermally induced chain aggregation [13]). Among the latter, hydrogels based on thermoresponsive poly(N-isopropylacrylamide) (PNIPAAm) are highly relevant due to their fast, reproducible, and easy-handled gelation process upon temperature increase, as PNIPAAm possesses a convenient lower critical solution temperature (LCST) of about 32 °C [14]. Nevertheless, PNIPAAm suffers several shortcomings. First, it provides insufficient water swelling and retention above the LCST due to its pronounced hydrophobicity, which leads to shrinkage of the gel and thus inadequate/uncontrolled properties. Secondly, PNIPAAm is non-degradable, which poses the problem of bioaccumulation at the injection site and possible further complications, which is a major barrier for clinical development. Intensive research has addressed these issues, using copolymerization approaches to introduce hydrophilic and/or degradable segments/moieties [15,16]. Such strategies typically consisted of either random copolymerization of NIPAAm with both a hydrophilic comonomer (e.g., poly(ethylene glycol) (di)methacrylate, 2-hydroxyethyl methacrylate) and a degradable one (oligolactide (meth)acrylate) [17,18,19,20], graft copolymerization from hydrophilic/degradable natural polymer backbone (alginate, hyaluronic acid) [21,22], or block copolymerization with polyethylene glycol (PEG) and aliphatic polyester segments (e.g., polylactide (PLA) and polycaprolactone (PCL)) [23,24]. Although suitable gelation properties are obtained, the mass loss observed during degradation studies remains generally limited. This is particularly due to the fact that the PNIPAAm-based residues produced upon degradation (i.e., polyester hydrolysis) remain mostly water insoluble, preventing their removal. Thus, the design of versatile PNIPAAm-based hydrogels able to exhibit degradation and bio-elimination features remains to be achieved to envision clinical development.

**Figure 1:**
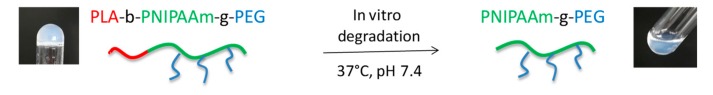
Description of the approach based on PLA-b-PNIPAAm-g-PEG, with PLA as a “sacrificial” block. PLA: polylactide, PNIPAAm: poly(N-isopropylacrylamide, PEG: polyethylene glycol.

In the present study, we have developed a PLA-b-PNIPAAm-g-PEG injectable hydrogel which upon PLA degradation (hydrolysis) loses the ability to gel as its PNIPAAm-g-PEG residues have a sufficiently high PEG content (Figure 1). Indeed, while PEG is highly desirable for bringing hydrophilicity in PNIPAAm-based hydrogels (to prevent water expulsion), it has been well documented that above a certain content, the gelation is not observed anymore under physiological conditions. This is due to the fact that the hydrophilic-PEG contribution readily counteracts the hydrophobically-induced aggregation of the PNIPAAm segments [25]. Under these conditions, we thus expected that the presence of the PLA block in the copolymer would promote gelation (by compensating the hydrophilic effect of PEG), and that its removal upon degradation would lead to soluble/dispersible PNIPAAm-g-PEG polymer prone to bio-elimination. We here successively describe the synthesis of the PLA-b-PNIPAAm-g-PEG architecture, its gelation behavior, degradation properties, and cytocompatibility, as a proof-of-concept study.

## 2. Experimental Section

### 2.1. Materials

Poly(ethylene glycol) methyl ether (MePEG, M_n_=2000 g/mol) was purchased from Sigma–Aldrich-Merck (Saint-Quentin Fallavier, France), D,L-Lactide (LA) was purchased from Corbion Purac (Amsterdam, The Netherlands), packaged under vacuum, and then stored under argon. Tin(II) 2-ethylhexanoate (SnOct_2_, 95%), anhydrous toluene, 2-hydroxyethyl acrylate (HEA), methacryloyl chloride (85%), 1,4-dioxane, N-Isopropylacrylamide, and dichloromethane (DCM) were also purchased from Sigma–Aldrich-Merck. Triethylamine (TEA) was purchased from Acros Organics (Illkirch, France), and BlocBuilder MA (MAMA-SG1) was provided by Arkema (Lacq, France). 1,4-dioxane and methacryloyl chloride were distilled before use.

### 2.2. Polymer Synthesis

#### 2.2.1. Synthesis of Polylactide-2-Hydroxyethyl Acrylate (PLA-HEA)

D,L-lactide (10.00 g, 69.38 mmol), HEA (287.7 mg, 2.48 mmol for a targeted degree of polymerization (DP) of 28) and SnOct_2_ (100 mg, 247 μmol) were placed in a single neck Schlenk, which was submitted to vacuum/argon cycles. Dry toluene (35 mL, [D,L-lactide] = 2 mol·L^−1^) was then added through the septum under an argon atmosphere in the Schlenk, which was placed in an oil bath preheated to 100 °C. After 2 h, the reaction was stopped by cooling the flask with ice for several minutes. The conversion was nearly total (96%), as determined from ^1^H NMR from the crude mixture. Toluene was removed under reduced pressure and the crude product diluted with THF. The reaction mixture was then precipitated in cold methanol to obtain pure PLA-HEA which was filtered, washed with methanol, and dried under vacuum.

#### 2.2.2. Synthesis of PLA-SG1 Macroinitiator/Alkoxyamine

PLA-HEA (3.9 g, 979 μmol) and BlocBuilder MA (3.7 g, 9.79 mmol) were introduced in a sealed vial. 8 mL of 1, 4-dioxane was added and the reaction mixture was deoxygenated by argon bubbling during 20 min. The vial was placed in an oil bath at 100 °C and the reaction mixture was stirred for 1 h, after which the reaction was stopped by cooling the vial with ice. The polymer was precipitated in excess cold methanol and dried under vacuum.

#### 2.2.3. Synthesis of Poly(Ethylene Glycol Methacrylate) (PEGMA) Macro-Monomer

MePEG (10 g, 5 mmol) was dissolved in dichloromethane (DCM) in a round-bottom flask and TEA (7 mL, 50 mmol) was added into the reaction mixture. Then methacryloyl chloride (5.2 g, 49.64 mmol) was added dropwise through an arm funnel at 0 °C under stirring. The reaction was allowed to run overnight at room temperature. The DCM was then removed under reduced pressure and the crude product was diluted with THF. After filtration of the TEA salts, the crude solution was precipitated in 90% diethyl ether and 10% ethanol mixture.

#### 2.2.4. Synthesis of PLA-b-P(NIPAAm-co-PEGMA) (i.e., PLA-b-PNIPAAm-g-PEG) Copolymer 

PLA-SG1 macroinitiator (0.1 g, 0.022 mmol), PEGMA (0.167 g, 0.0835 mmol) and NIPAAm (0.85 g, 7.5 mmol) were placed in a sealed vial and 5 mL of 1, 4-dioxane were added into the vial. The reactants were dissolved and degassed for about 20 min. The mixture was then immersed in an oil bath at 120 °C and stirred for 42 h. The crude product was precipitated twice in excess cold diethyl ether, filtered and dried under vacuum (conversions: 90% in NIPAAm, 100% in PEGMA). The P(NIPAAm-co-PEGMA) copolymer as control was obtained under the same procedure except that BlocBuilder MA (8.4 mg, 0.022 mmol) was used as an initiator instead of PLA-SG1.

### 2.3. Polymer Characterization

^1^H NMR spectra were recorded on a Bruker Advance 400 MHz spectrometer (Bruker, Karlsruhe, Germany) to assess compositions, end-chain functionalizations and degradation extent. The solvents used were deuterated chloroform (CDCl_3_) and deuterated dimethyl sulfoxide (DMSO-d6).

Size exclusion chromatography (SEC) experiments were performed for determination of molecular weights and dispersity on a PL-GPC 120 apparatus (Agilent, Santa Clara, CA, USA) which was composed of a PL-AS-MT autosampler, an Agilent 1100 series pump, a degasser, an injection valve, a column oven, and a refractive index (RI) detector. The following columns were used: one pre-column and two PL Resipore columns (300 mm A~7.8 mm). The injection loop, the columns, and the RI detector were in the same oven thermostated at 70 °C. The eluent was a solution of 0.1 M LiBr in N,N-dimethylformamide (DMF) filtered through a 0.45 μm nylon membrane and the flow rate was fixed at 0.7 mL·min^−1^. The samples were prepared in a mixture of eluent and toluene (0.25 vol%) as the flowmarker, filtered through a 0.2 μm nylon filter (Interchim), and placed in an autosampler preheated to 50 °C. The sample concentration was 0.25 wt. %. Calibration curves were established with poly(methyl methacrylate) (PMMA) standards purchased from Agilent. 

### 2.4. Copolymer Aqueous Solution Properties vs. Temperature

#### 2.4.1. Dynamic Light Scattering (DLS)

DLS analysis was performed on 0.1 wt. % copolymer solutions in phosphate buffer saline (PBS) (pH 7.4). The hydrodynamic diameter of the micelles as a function of temperature was measured using a Zetasizer Nano ZS apparatus (Malvern, UK). After equilibration at 20 °C, the temperature was incremented (in steps of 1 or 2 °C) to 43 °C. For each step, an equilibration period was fixed at 3 min before performing 2 measurements.

#### 2.4.2. UV-visible Measurements with Cyanine 5.5-Carboxyl Probe

20 µL of acetonic solution of cyanine 5.5-carboxyl (Cy5.5, FluoProbes) at 0.35 mg·mL^−1^ was introduced in 2 test tubes and the acetone evaporated under a hood for 3 h. Then 2.1 mL of PLA-b-P(NIPAAm-co-PEGMA) or P(NIPAAm-co-PEGMA) solutions (0.1 wt. % in PBS) were added to each tube of Cy5.5, and the solutions were stirred for 2 h. Then, the solutions were transferred into plastic cuvettes for UV-visible measurements from 630 to 750 nm (Cary 50 Varian spectrometer, Agilent).

#### 2.4.3. Inverted Tube Method

The inverted test tube method was used to determine the sol-gel transition phase of PLA-b-P(NIPAAm-co-PEGMA) and P(NIPAAm-co-PEGMA) in PBS solution at 10 or 15 wt. %. The content in each vial was made homogenous by proper mixing and stored at 4 °C for 5 days for complete dissolution. Both samples were heated from 10 °C to 55 °C and photographs were taken.

### 2.5. Degradation Studies

Hydrogel degradation kinetics was investigated in physiological conditions at 37 °C in PBS pH 7.4. Typically, the PLA-P(NIPAAm-co-PEGMA) block copolymer (50 mg) was dissolved in PBS (0.33 mL, i.e., 15 wt. % copolymer concentration) in a 5-mL test tube and set at 37 °C until complete gelation. Then, 2.5 mL of PBS (pre-heated at 37 °C) was added to the tube, and incubated at 37 °C. At predetermined times, a volume of sample was withdrawn, dried under vacuum and analyzed by ^1^H NMR analysis in DMSO-d6.

### 2.6. Cell Culture Protocol

The cell line used was a murine dendritic cell line (DC 2.4) and was grown according to typical culture procedure detailed here. The cell culture media was composed of RPMI-1640, Fetal Bovine Serum (FBS) (10%), 2-mercaptoethanol (50 µM), and 4-(2-hydroxyethyl)-1-piperazineethanesulfonic acid (HEPES) buffer solution (10 mM). After aspirating the old culture media, the cells which adhered to the bottom of the flask T75 were washed twice with 10 mL of PBS. Then, 1 mL of trypsin was added to the cells and left for 3 min at 37 °C. After 3 min, the trypsin solution containing the cells was mixed with 9 mL of fresh complete culture media. The appropriate number of cells was re-suspended in 13 mL of fresh culture media in a new flask T75, and left in the CO_2_ incubator at 37 °C. Cells were used with a low passage number (less than 10).

### 2.7. Cytotoxicity Studies

Cytotoxicity was evaluated by both MTT assay and Presto Blue Assay (Thermo Fisher Scientific, Waltham, MA, USA, according to the manufacturer’s instructions. Briefly, DC2.4 cells were seeded at a density of 20,000 cells/well (in 100 µL of complete medium) into 96-well plates a day prior to incubation with the copolymer (one plate for each test). A stock solution of copolymer at 1.5 mg·mL^−1^ in DPBS (Dulbecco’s PBS) was prepared, from which samples of concentrations ranging from 7.5 to 150 µg·mL^−1^ were prepared from appropriate dilution in complete medium. After aspirating the complete medium from the wells, 100 µL of each sample concentration was added to the cells (in triplicate). Control samples were performed by using DPBS solution instead of stock solution of copolymer using the corresponding volumes. For both tests, blanks were also performed without cells, to check any interference of the copolymer with the test used. After incubation for 20 h, treatments with the convenient reagents were performed according to the manufacturer’s instructions. Absorbance was measured at 570 nm for MTT assay with a Tecan i-control Infinite M1000, and fluorescence emission was measured at 590 nm using the same apparatus (excitation wavelength: 560 nm) for Presto Blue assay.

## 3. Results and Discussion

### 3.1. Polymer Synthesis

Our block copolymer relied on 3 compounds that provided complementary properties: PNIPAAm for the thermosensitive (i.e., injectable) character, PLA for degradability, and PEG for its hydrophilicity ensuring water retention. As mentioned earlier, our PLA-b-PNIPAAm-g-PEG block architecture design was expected to lead, after PLA hydrolysis, to a polymer unable to gel at physiological temperature, providing sufficient PEG content.

**Scheme 1:**
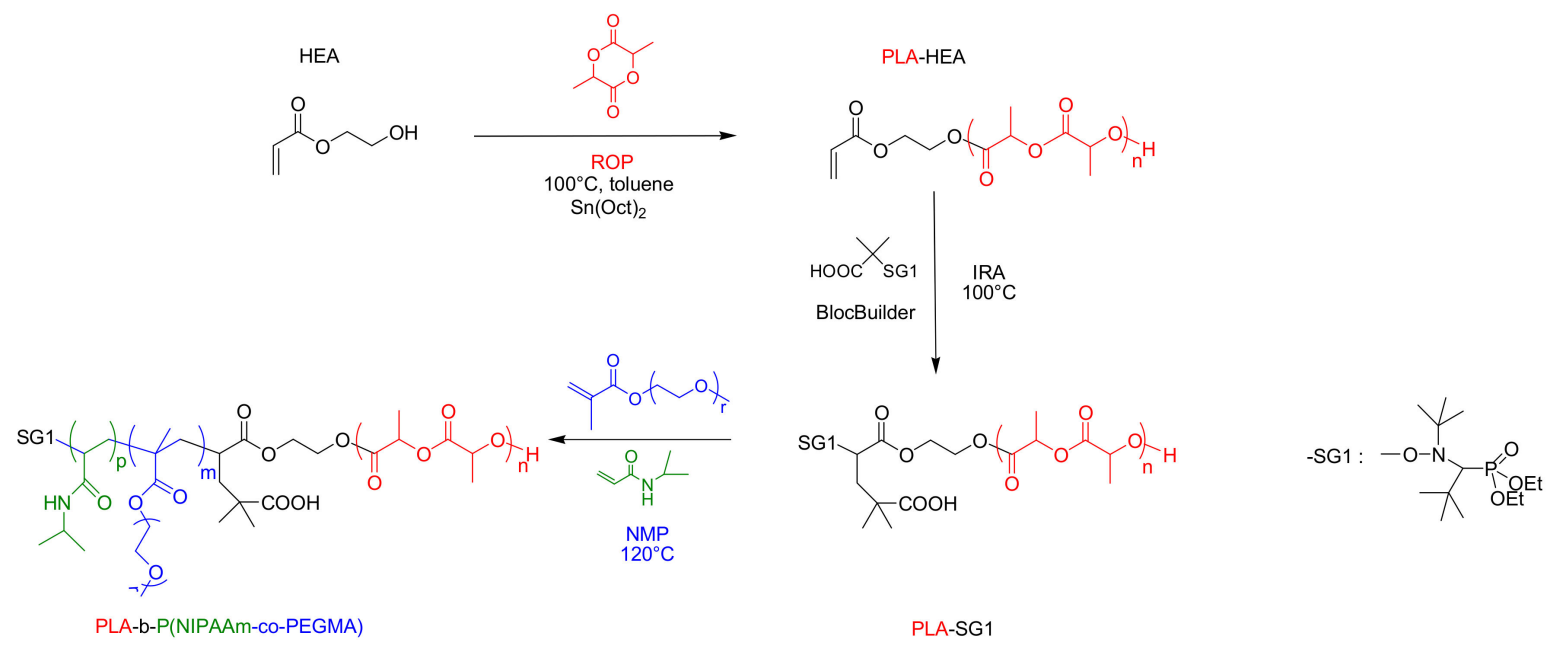
Preparation scheme of the poly(D,L-lactide)-b-poly(NIPAAm-co-polyethylene glycol methacrylate) (PLA-b-P(NIPAAm-co-PEGMA)) copolymer.

The synthesis of the PLA-b-PNIPAAm-g-PEG copolymer (referred to as PLA-b-P(NIPAAm-co-PEGMA) in the rest of the paper) relied on a combination of ring-opening polymerization (ROP) and nitroxide-mediated polymerization (NMP). In the first step, an acrylate-terminated PLA (PLA-HEA) was obtained by performing ROP of LA from 2-hydroxyethyl acrylate (HEA) as the initiator in the presence of SnOct_2_ as the catalyst. Then, the BlocBuilder alkoxyamine was added onto the acrylate double bond of the PLA-HEA through intermolecular radical addition (IRA). This step yielded a PLA-SG1 macroalkoxyamine, able to initiate the NMP of NIPAAm and PEGMA, leading to the formation of PLA-b-P(NIPAAm-co-PEGMA) copolymer (Scheme 1). 

**Figure 2:**
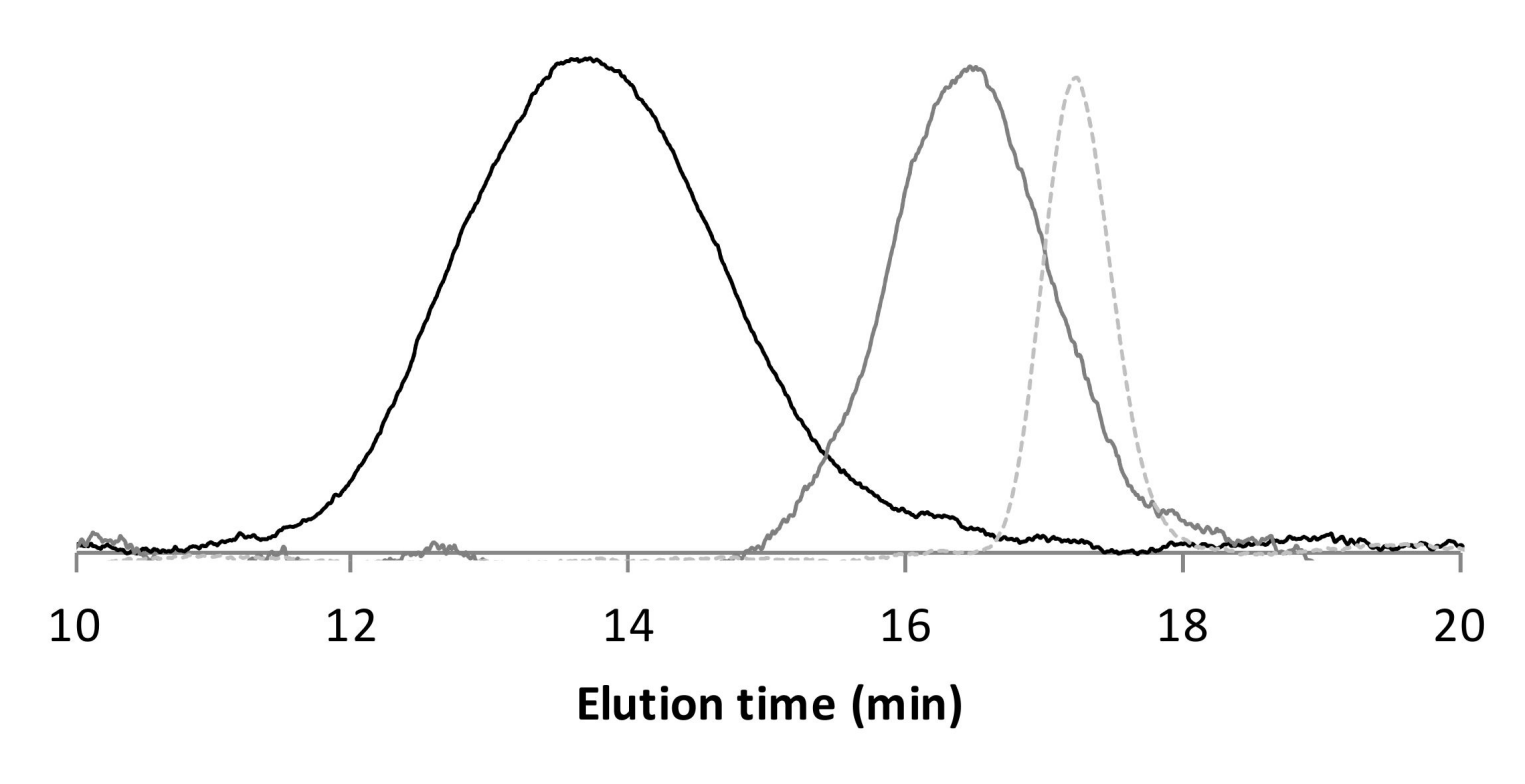
Size exclusion chromatography (SEC) analysis (in N,N-dimethylformamide (DMF)) of the (co)polymers along synthesis process. From right to left: poly(ethylene glycol) methacrylate (PEGMA) (grey dotted line): *M*_n_(SEC) = 3900 g·mol^−1^, Đ = 1.06; PLA-SG1 (grey line): *M*_n_(SEC) = 5700 g·mol^−1^, Đ = 1.36; PLA-b-P(NIPAAm-PEGMA) (black line): *M*_n_(SEC) = 46,000 g·mol^−1^, Đ = 1.9.

The polymers obtained from each step (after precipitation) were analyzed by ^1^H NMR. The spectrum of PLA-HEA revealed the presence of the proton signals from both chain ends (acrylate and CO–(C*H*)CH_3_OH) with integration consistent with a nearly total initiation efficiency of the HEA (Figure S1, Supplementary Information). The degree of polymerization (DP) determined from integration (DP = 33, *M*_n_ = 4700 g·mol^−1^) was in good agreement with the targeted one (DP = 28). After IRA of the MAMA-SG1 alkoxyamine, vinyl protons of the HEA completely disappeared while t-butyl proton singlets of the SG1 nitroxide (around 1.0 ppm, 9H + 9H) were observable with integration consistent with nearly a total functionalization yield (99%, Figure S2). Finally, the spectrum of the block copolymer obtained after NMP of NIPAAm and PEGMA showed characteristic proton peaks from the PLA, PNIPAAm, and PEG segments, and composition could be determined from integration (Figure S4). A weight composition of 7/77.5/15.5 wt. % in PLA/PNIPAAm/PEG was determined, close to the feed ratios (9/76/15). Peak shifting was observed for the copolymer by SEC analysis as compared to that of PLA-SG1 precursor, with neither residual PLA-SG1 nor PEGMA macromer (Figure 2), which, combined with the NMR results, was indicative of the formation of the desired block copolymer. The block copolymer molecular weight from SEC was *M*_n_ = 46,000 g·mol^−1^ (with a dispersity of 1.9). This *M*_n_ value was well-compatible with a possible renal excretion, and even more considering that molecular weight was expected to further decrease after PLA hydrolysis.

It is important to note here that this copolymer composition was found optimal regarding gelation (see later results) based on preliminary screening synthesis efforts. Indeed PLA/PEG ratio had to be carefully adjusted—sufficiently high so that hydrophobic PLA can counteract the effect of PEG hydrophilicity for gelation process below 37 °C, but also low enough to preserve the beneficial impact of hydrophilic PEG for water uptake and retention. On the other hand, PNIPAAm content had to be high enough to ensure gelation process through hydrophobically induced aggregation. For further investigations, the same polymer without PLA block (i.e., P(NIPAAm-co-PEGMA)) was synthesized as a control, using MAMA-SG1 as an initiator instead of PLA-SG1 (in same molar amounts). The PEGMA/PNIPAAm relative composition weight ratio determined from ^1^H NMR integration was the same as for the PLA-b-P(NIPAAm-co-PEGMA) (Figure S5), namely 1/5.

### 3.2. Aqueous Solution Properties of the Copolymer

**Figure 3:**
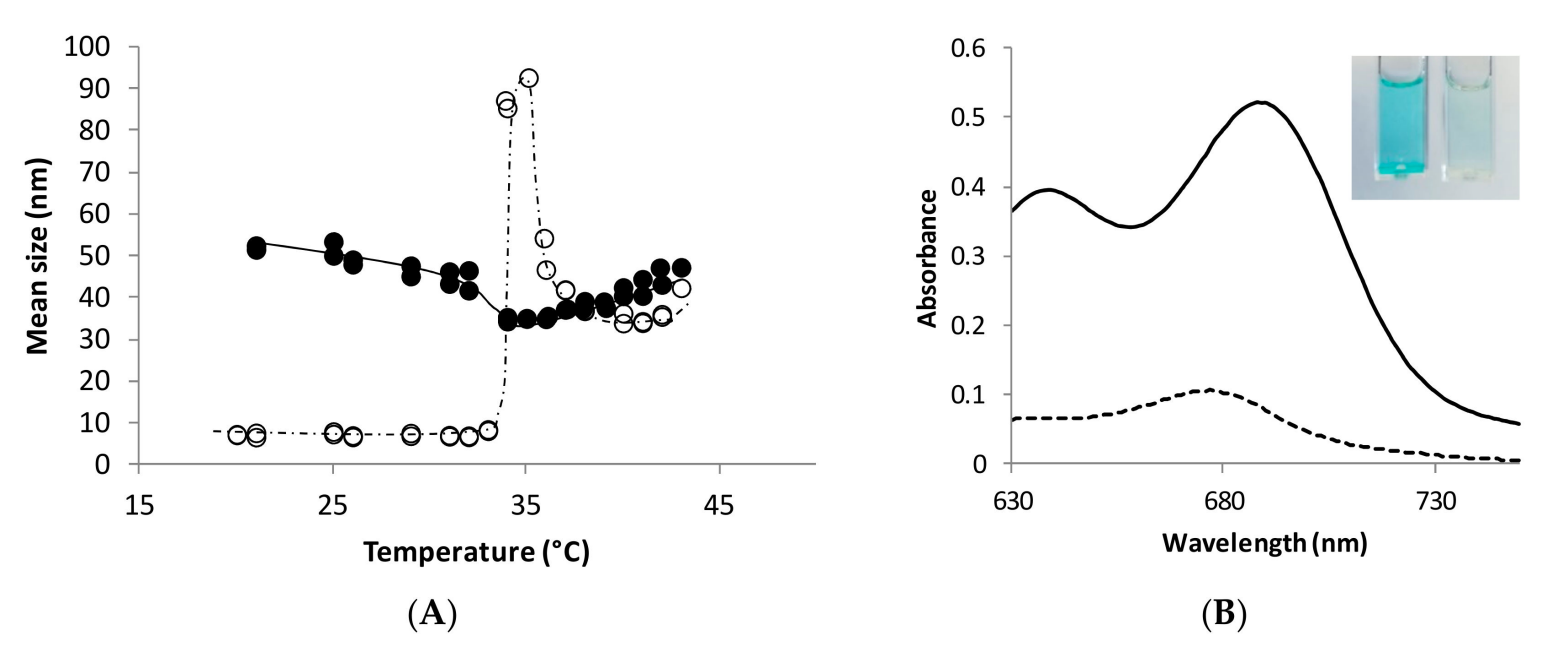
Aqueous solution behavior of the copolymers in phosphate buffer saline (PBS) (0.1 wt. %): (**A**) size (from dynamic light scattering (DLS)) vs. temperature for PLA-b-P(NIPAAm-co-PEGMA) (●) and P(NIPAAm-co-PEGMA) (○); (**B**) UV-visible spectrum of cy5.5 incubated at room temperature with copolymer solutions (Cy5.5: 3.3 µg·mL^−1^, copolymer: 1°mg·mL^−1^ (0.1 wt. %) in PBS): PLA-b-P(NIPAAm-co-PEGMA) (back line, left photograph) and P(NIPAAm-co-PEGMA) (dotted line, right photograph).

Aqueous solution behavior of copolymer with PLA, (PLA-b-P(NIPAAm-co-PEGMA)) and without PLA, (P(NIPAAm-co-PEGMA) was investigated in diluted state by DLS (0.1 wt. % of copolymer in filtered PBS (i.e., 1 mg·mL^−1^)) as a function of temperature. Below transition temperature of PNIPAAm (i.e., when it is water-soluble), the behavior was different for the two copolymers (Figure 3A). Indeed, P(NIPAAm-co-PEGMA), since entirely water soluble, exhibited size of less than 10 nm (representative of individual chains in solution) while PLA-P(NIPAAm-co-PEGMA) polymer self-assembled in micelles of about 50 nm due to its amphiphilic character (PLA hydrophobic segment as core). Upon heating above 30 °C, the size of the PLA-P(NIPAAm-co-PEGMA) micelles decreased to about 35 nm, because the PNIPAAm segment becomes hydrophobic and tends to move into the core of the micelles. As for P(NIPAAm-co-PEGMA), micelles (~35 nm) were formed upon heating above 30 °C (after a brief destabilization phase) as the copolymer becomes amphiphilic, due to the PNIPAAm hydrophilic-to-hydrophobic transition. The existence of a micellar state for PLA-b-P(NIPAAm-co-PEGMA) at room temperature was supported by hydrophobic dye (cyanine 5.5 carboxyl, Cy5.5) solubilization. Indeed, PBS solution of the copolymer (0.1 wt. %) mixed with Cy5.5 (3.3 μg·mL^−1^) led to significant solubilization of the dye (through micellar encapsulation) while the dye remained mostly insoluble in the case of the control P(NIPAAm-co-PEGMA) copolymer, as shown by UV-visible absorption (Figure 3B). In addition to strong absorbance increase, the encapsulation of the dye provoked a shift of the λ_max_ toward higher wavelengths as a result of environmental change of the dye [26] (i.e., from 677 nm in aqueous medium to 690 nm in micelle core), supporting that Cy5.5 was located in the hydrophobic core (PLA) of the micelles. Together with DLS, this clearly showed that PLA in the copolymer induces amphiphilic character for PLA-b-P(NIPAAm-co-PEGMA) and thus micelle formation. It is also interesting to note that the LCST of the PLA-b-P(NIPAAm-co-PEGMA) was slightly lower (32 °C) than that of the P(NIPAAm-co-PEGMA) control (34 °C). This was in good accordance with previous data, which show a decrease of LCST induced by hydrophobic segments such as PLA [17,18].

**Figure 4:**
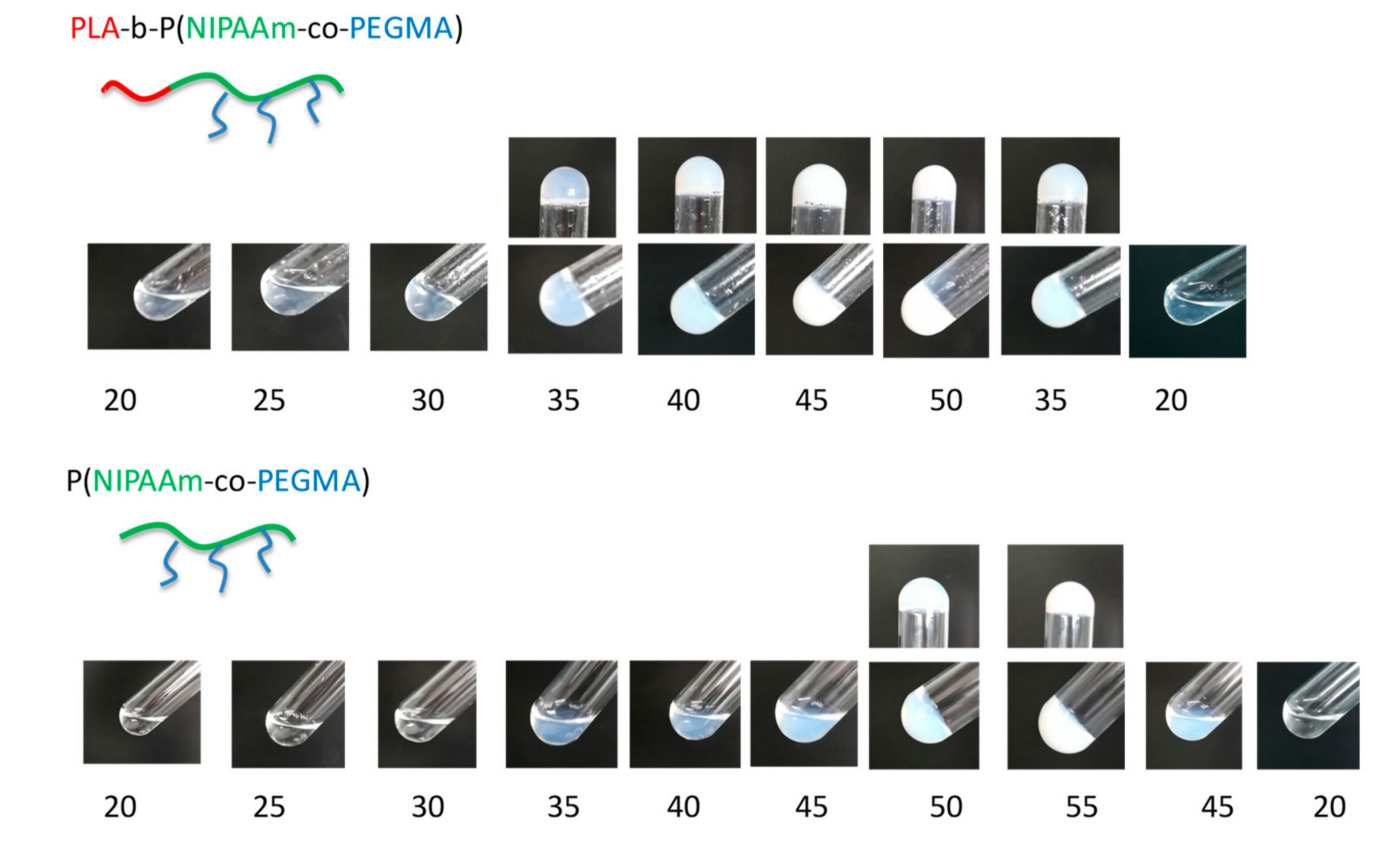
Hydrogel formation vs. temperature (copolymer at 15 wt. % in PBS) through inverted tube method: PLA-b-P(NIPAAm-co-PEGMA) (top) and P(NIPAAm-co-PEGMA) (bottom).

The sol-gel transition of concentrated solution of PLA-b-P(NIPAAm-co-PEGMA) in PBS (15 wt. %) was further investigated (Figure 4, top) by the inverted tube method. Gel formation was observed around 30–35 °C. Such a property was highly suitable for further in vivo injectability as gelation occurs below 37 °C, but not below 30 °C, meaning that the copolymer solution can be conveniently handled in liquid state at room temperature. Interestingly, in the case of the P(NIPAAm-co-PEGMA) control without PLA (Figure 4, bottom), the gel formation occurred at a higher temperature (50 °C), although turbidity increase (associated with the transition of the PNIPAAm segment) began to be observed at ~35 °C, consistent with the previous DLS results. Thus, PLA was clearly responsible for lowering the gelation temperature of PLA-b-P(NIPAAm-co-PEGMA). These results supported the relevance of our approach, namely that upon PLA hydrolysis, the forming copolymer will be unable to gel anymore at physiological temperature, and thus be potentially removable. Based on DLS results, we could speculate that gel formation for the PLA-b-P(NIPAAm-co-PEGMA) copolymer was closely linked to the pre-existence of micelles at room temperature and their further rearrangement following the hydrophilic-hydrophobic transition of PNIPAAm segments upon heating. To note, PLA-b-P(NIPAAm-co-PEGMA) at lower concentration in PBS (10 wt. %) did not turn into a gel in physiological conditions, showing that the concentration has to be sufficiently high to induce efficient PNIPAAm entanglement.

### 3.3. Hydrogel Degradation Studies

**Figure 5:**
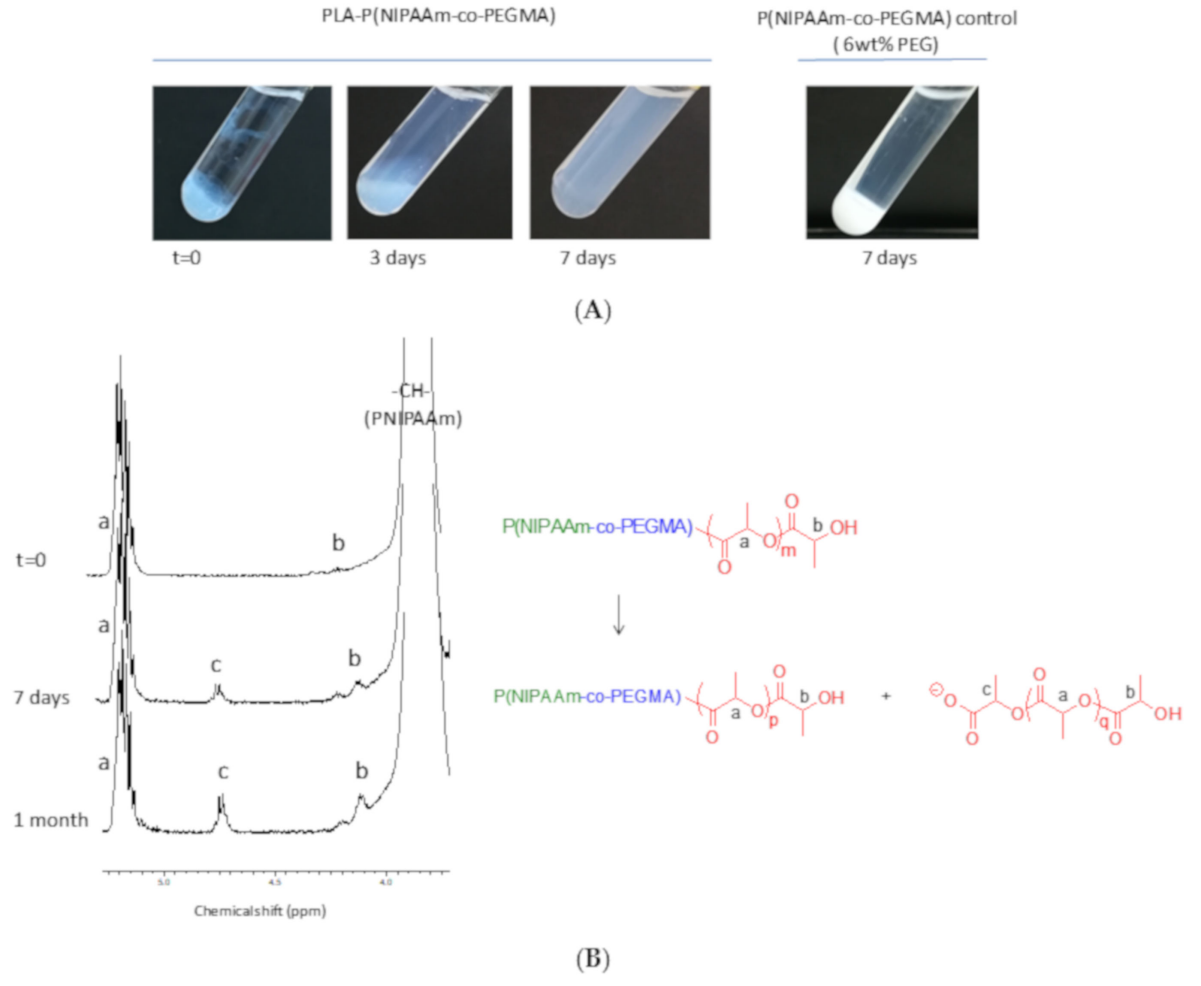
In vitro degradation (PBS, pH 7.4, 37 °C) of PLA-P(NIPAAm-PEGMA) hydrogel, assessed visually (**A**, photographs) and quantitatively (**B**, ^1^H NMR in deuterated dimethyl sulfoxide (DMSO-d6)).

Degradation of the hydrogel (15 wt. % copolymer in PBS) was investigated in standard physiological conditions. (37 °C, PBS pH 7.4). The hydrogel gradually eroded until complete disappearance and mass loss in 7 days, yielding a final homogeneous and slightly turbid solution at 37 °C (Figure 5A). The latter was analyzed by ^1^H NMR in DMSO-d6 after drying (Figure 5B, middle spectrum). Interestingly, the spectrum reveals a significant presence of the proton signals characteristic of the methine protons (*c*, *b*) adjacent to the −COOH and −OH lactide end groups (4.85 and 4.25 ppm, respectively), as compared to the initial intact block copolymer (presenting only −CH-lactide proton signal, 5.2 ppm, *a*). This evidenced the hydrolysis of the ester bonds along the PLA block, leading to cleavage of the block copolymer. Based on integration showing a signal ratio of protons a to protons c of about 15, it was found that oligolactide residues of maximum 1000 g·mol^-1^ were formed, assuming random scission along the chain. Thus, our copolymer upon PLA hydrolysis at 37 °C tended progressively to approach the structure of P(NIPAAm-co-PEGMA), unable to gel at this temperature (Figure 4, bottom), and thus released in PBS surrounding medium. Oligolactide cleavage through ester hydrolysis was shown to further continue, as shown on the spectrum obtained for the dispersion at longer times (bottom spectrum, Figure 5B), by the increase of both proton signals *b* and *c*.

To support that the hydrogel mass loss was uniquely due to PLA scission and no other eventual effects, we performed a control experiment using a P(NIPAAm-co-PEGMA) copolymer able to gel at 37 °C, namely a P(NIPAAm-co-PEGMA) copolymer containing a reduced content in PEG (~6 wt. %). In the same conditions as in the present study (15 wt. % copolymer in hydrogel, surrounding PBS medium), absolutely no release was detected in the surrounding PBS, the entire hydrogel mass remaining localized at the bottom of the test tube (Figure 5A, right).

### 3.4. Cytotoxicity Evaluation

**Figure 6:**
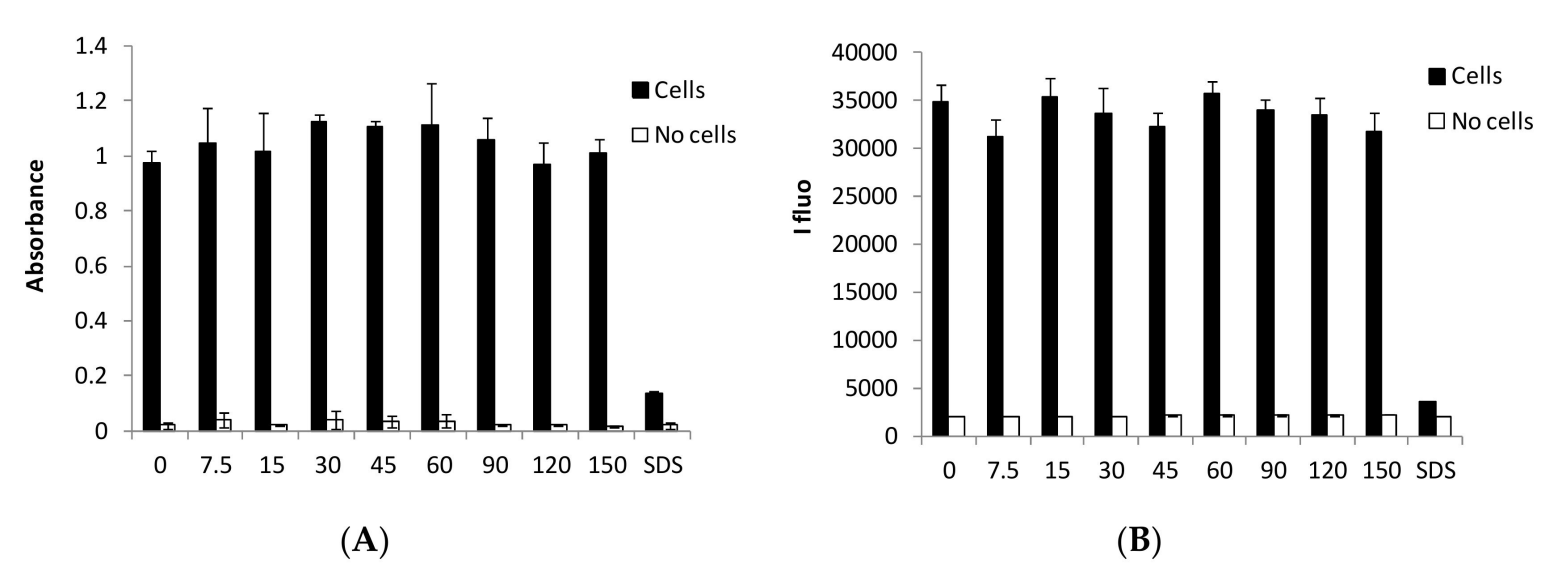
Cytotoxicity of the PLA-b-P(NIPAAm-co-PEGMA) copolymer towards dendritic cells (DC 2.4) at various concentrations (7.5 to 150 µg·mL^−1^) assessed by MTT (**A**) and Presto Blue (**B**) assays. “0” means no polymer (signal of cell alone is the one on which the results are normalized to calculate cell viability). Sodium dodecyl sulfate (SDS) (right) was used as a toxic positive control.

Cytotoxicity of the copolymer was evaluated on dendritic cells (DC 2.4) which are known to be particularly sensitive to toxicity and are widely used in studies with vaccine-delivery perspectives. The cytotoxicity was evaluated in a classical manner by assessing the metabolic activity of the cells through their ability to reduce a compound to another, easily detectable by spectroscopy (UV-visible or fluorescence). As shown in Figure 6A for the MTT test (3-(4,5-dimethylthiazol-2-yl)-2,5-diphenyltetrazolium bromide), the copolymer showed no toxicity whatever the concentration range used (representative of in vivo context) with a cell viability approaching 100%. Importantly, we carefully checked that no UV interference occurred between MTT reagent and the polymer in absence of cells (“no cells”, Figure 6A), as MTT was previously reported to be potentially sensitive to certain species, leading to non-negligible absorbance and thus non-relevant results [27]. The non-toxicity of the copolymer was further fully confirmed by the resazurin-based Presto Blue assay (Figure 6B), which showed similar cell viability of nearly 100% over the same concentration range. These cytotoxicity results were highly consistent with previously reported data displaying absence of toxicity of PNIPAAm-based copolymers, particularly with PLA and PEG compounds [24,28,29,30]. They validate the synthesis scheme and design of this new block copolymer for bio-related applications.

## 4. Conclusions

In this paper, we developed a new approach of a PLA/PNIPAAm/PEG injectable copolymer hydrogel relying on a “sacrificial” PLA block, leading upon hydrolysis to a non-gelated and dispersible polymer, for bio-elimination purpose. The copolymer was obtained in a versatile and well-defined manner by combination of ROP, IRA, and NMP. The PLA hydrophobic block was responsible for the existence of micelles at room temperature due to amphiphilicity, and successful gelation at 37 °C at 15 wt. % concentration. It was demonstrated that gelling properties were lost due to hydrolysis of the PLA block, leading to a non-gelated and dispersible PNIPAAm/PEG part due to important hydrophilic PEG contribution. This novel copolymer was shown to be non-toxic. This validated concept opens new avenues for designing degradable/bio-eliminable PNIPAAm hydrogels. Further studies will focus on rheological, injectable, and morphological properties of such new hydrogel platform as well as their tunability, in the perspective of bio-related applications.

## Supplementary Materials

The following are available online at https://www.mdpi.com/2073-4360/12/4/925/s1, Figure S1: ^1^H NMR spectrum of PLA-HEA (CDCl_3_, 400 MHz), Figure S2: ^1^H NMR spectrum of PLA-SG1 (CDCl_3_, 400 MHz), Figure S3: ^1^H NMR spectrum of PEGMA (CDCl_3_, 400 MHz), Figure S4: ^1^H NMR spectrum of PLA-b-P(NIPAAm-co-PEGMA) (CDCl_3_, 400 MHz), Figure S5: ^1^H NMR spectrum of P(NIPAAm-co-PEGMA) (CDCl_3_, 400 MHz).
