# Supplementary material for: A Sacrificial PLA Block Mediated Route to Injectable and Degradable PNIPAAm-Based Hydrogels
Source: Polymers (Basel). 2020 Apr 16;12(4):925. doi: 10.3390/polym12040925 (PMC7240404; doi:10.3390/polym12040925)
Supplement: Supplementary file 1 [file polymers-12-00925-s001.pdf]

Electronic Supplementary Information

A sacrificial PLA block mediated route to injectable and degradable PNIPAAm based hydrogels

Vernon Tebong Mbah[1,2], Vincent Pertici[1], Pierluigi Stipa[2], Bernard Verrier[3], Céline Lacroix[3], Didier Gigmes[1], Thomas Trimaille[1*]

[1] Aix Marseille Univ, CNRS, ICR, Marseille, France

[2] Dipartimento di Scienze dei Materiali e della Terra, Università Politecnica delle Marche, via Brecce Bianche, 60131 Ancona, Italy

[3] Université Claude Bernard Lyon 1, Laboratoire de Biologie Tissulaire et d'Ingénierie Thérapeutique, UMR 5305, Université Lyon 1, CNRS, IBCP, 69367 Lyon, France

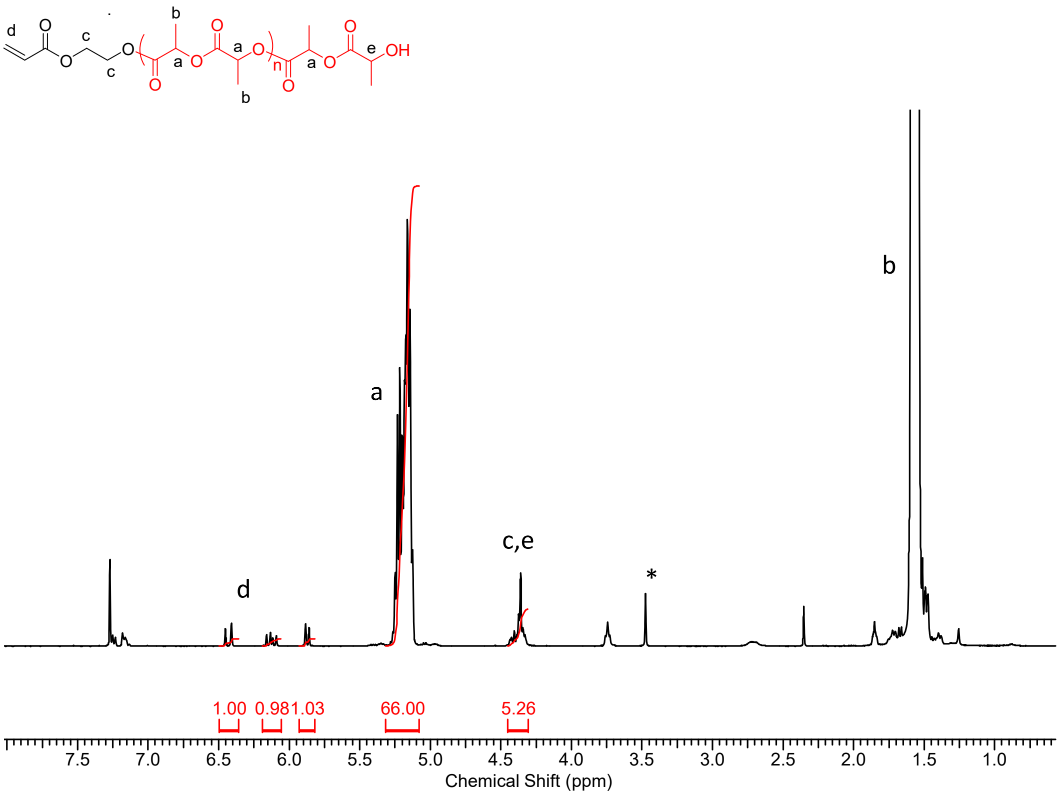

Figure S1. ^1H NMR spectrum of PLA-HEA ($CDCl_3$, 400 MHz), *: methanol

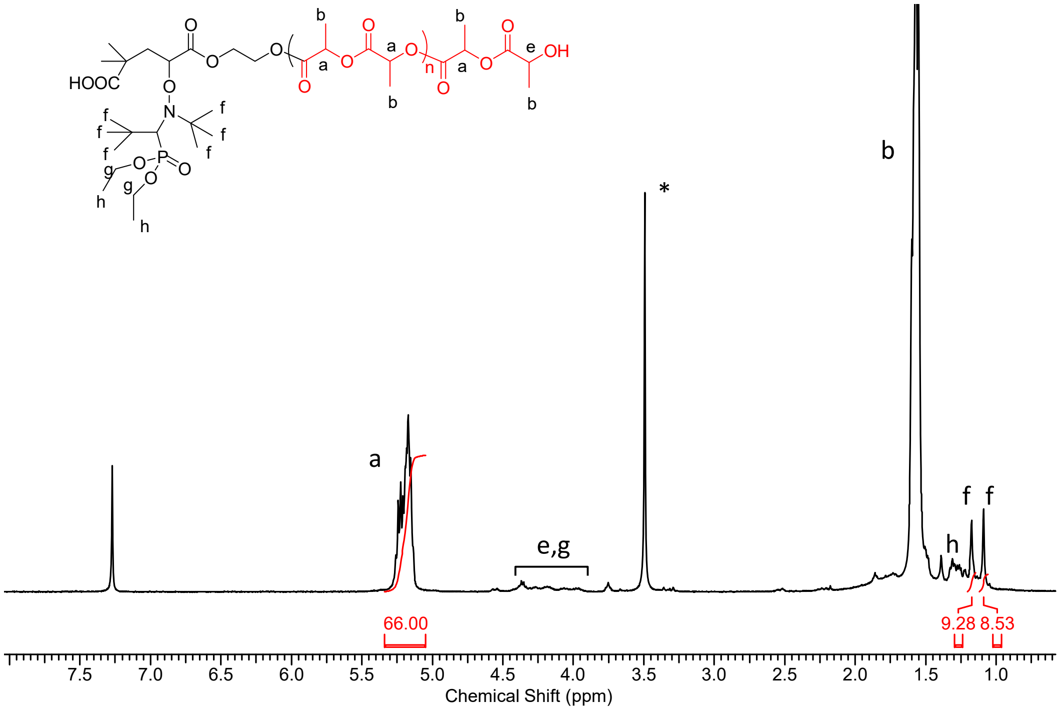

Figure S2.¹H NMR spectrum of PLA-SG1 ($CDCl_3$, 400 MHz), *: methanol

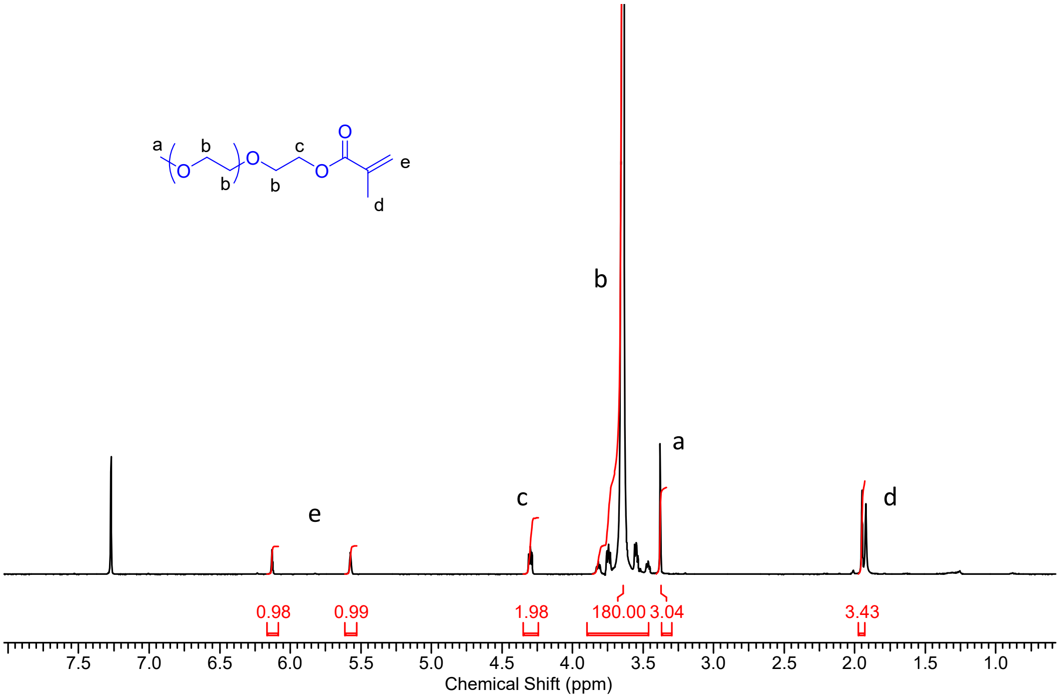

Figure S3. ¹H NMR spectrum of PEGMA ($CDCl_3$, 400 MHz)

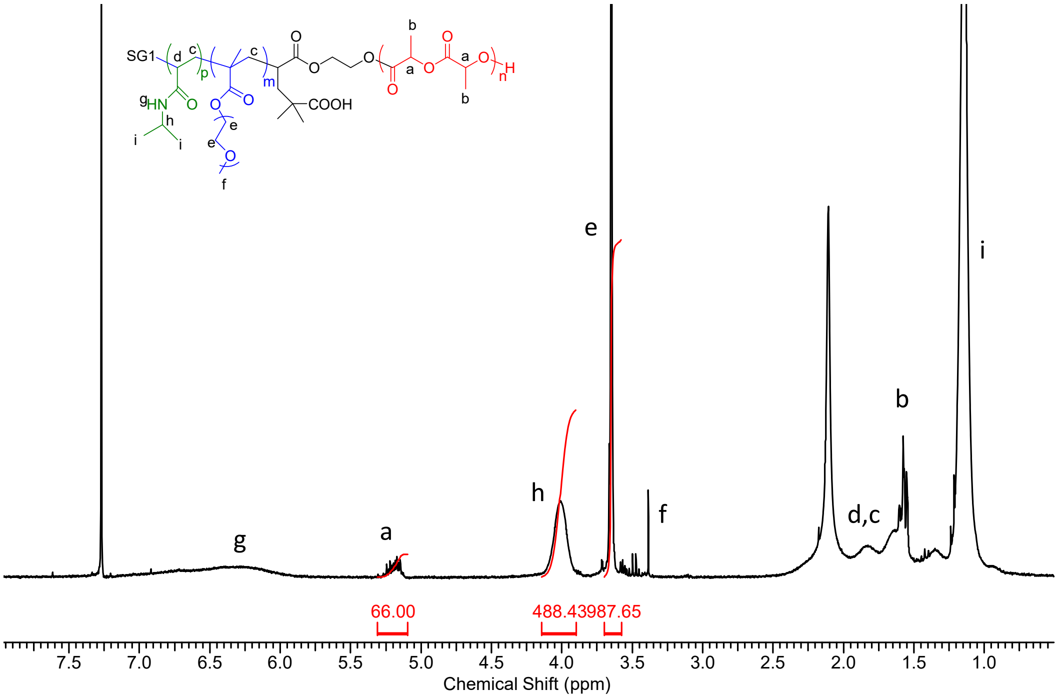

Figure S4. ^{1}H NMR spectrum of PLA-b-P(NIPAAm-co-PEGMA) ($CDCl_3$, 400 MHz)

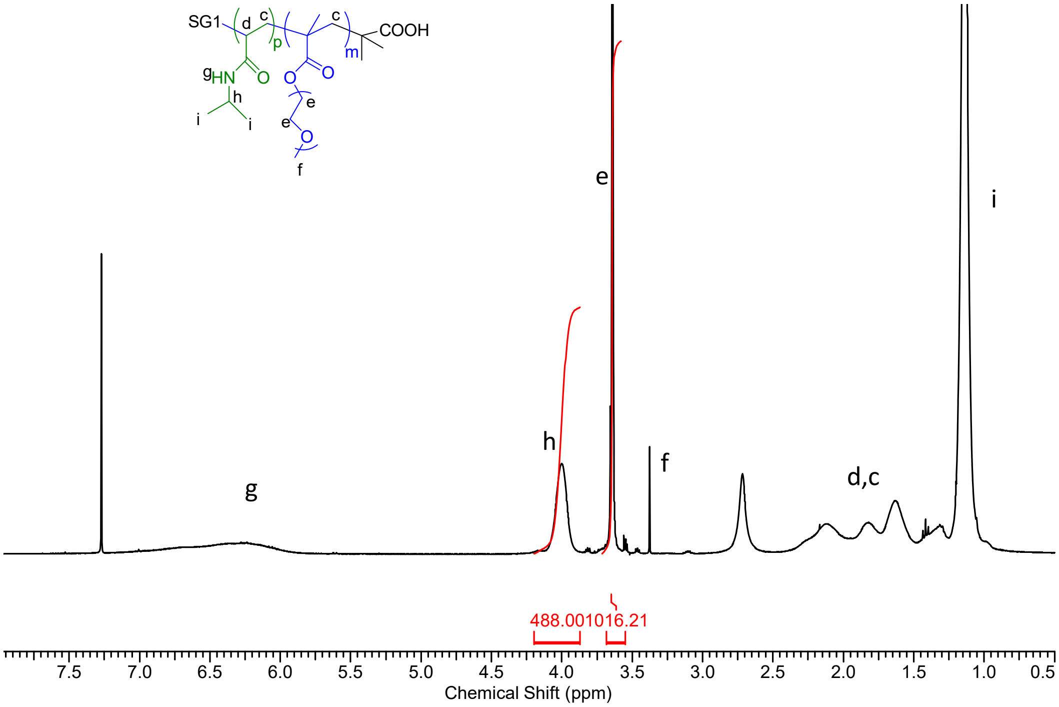

Figure S5. ^{1}H NMR spectrum of P(NIPAAm-co-PEGMA) ($CDCl_3$, 400 MHz)
